# Supplementary material for: Electrical and Structural Properties of Semi-Polar-ZnO/a-Al2O3 and Polar-ZnO/c-Al2O3 Films: A Comparative Study
Source: Materials (Basel). 2022 Dec 23;16(1):151. doi: 10.3390/ma16010151 (PMC9821142; doi:10.3390/ma16010151)
Supplement: Supplementary file 1 [file materials-16-00151-s001.zip › materials-2036403-supplementary.pdf]

Supplementary Materials

Electrical and Structural Properties of Semi-polar-ZnO/*a*-Al_2O_3 and Polar-ZnO/c-Al_2O_3 ZnO Films: A Comparative Study

Sushma Mishra, Wojciech Paszkowicz, Adrian Sulich, Rafal Jakiela, Monika Ożga and Elżbieta Guziewicz *

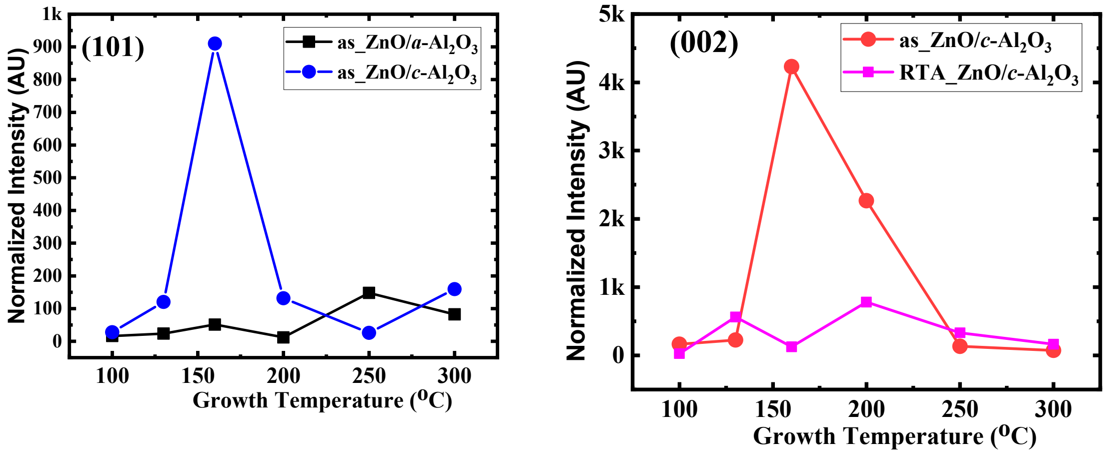

Figure S1. Normalized intensity variation of XRD peaks (integrals) with growth temperature (a) as grown and (b) annealed, $ZnO_{(101)}$/*a*-Al_2O_3 and $ZnO_{(002)}$/*c*-Al_2O_3 films

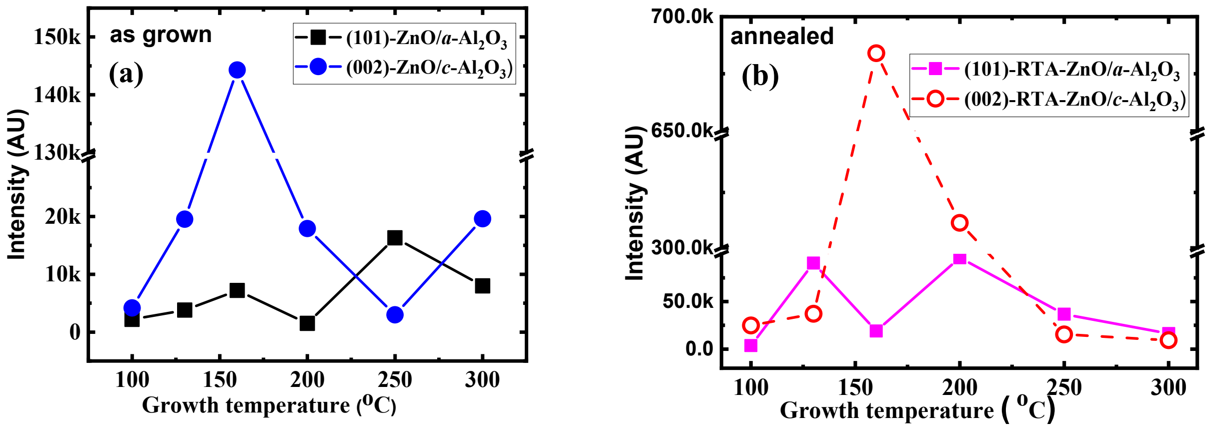

Figure S2. Intensity variation of XRD peaks (integrals) with growth temperature (a) as grown and (b) annealed, $ZnO_{(101)}$/*a*-Al_2O_3 and $ZnO_{(002)}$/*c*-Al_2O_3 films

Table S1. Crystallites size variation with T_g, for as grown and annealed ~150nm ZnO films on *a*-Al_2O_3 and *c*-Al_2O_3 substrates.

T_g degC	Grain(nm) (101)_as *a*-Al2O3	Grain(nm) (101)_RTP *a*-Al2O3	Grain(nm) (002)_as *a*-Al2O3	Grain(nm) (002)_RTP *a*-Al2O3	Grain(nm) (002)_as *c*-Al2O3	Grain(nm) (002)_RTP *c*-Al2O3	Grain(nm) (101)_as *c*-Al2O3	Grain(nm) (101)_RTP *c*-Al2O3
**100**	12.03	38.55	23.19	-	24.75	37.53	-	-
**130**	14.41	41.72	23.22	-	28.19	48.64	-	-
**160**	14.32	37.41	46.1	62.34	36.21	85.06	-	-
**200**	14.49	45.23	-	-	13.07	51.22	-	-
**250**	15.47	37.79	8.97	-	9.38	14.54	65.68	-
**300**	19.25	43.13	15.92	-	70.44	70.88	-	-

Table S2 Micro-strain variation with T_g, for as grown and annealed ~150nm ZnO films on *a*-Al_2O_3 and *c*-Al_2O_3 substrates.

T_g degC	microstrain (101) as_(10^{-3}) *a*-Al2O3	microstrain (101) RTP_(10^{-3}) *a*-Al2O3	microstrain (002) as_(10^{-3}) *a*-Al2O3	microstrain (002) RTP_(10^{-3}) *a*-Al2O3	microstrain (101) as_(10^{-3}) *c*-Al2O3	microstrain (101) RTP_(10^{-3}) *c*-Al2O3	microstrain (101) as_(10^{-3}) *c*-Al2O3	microstrain (101) RTP_(10^{-3}) *c*-Al2O3
**100**	1.05	2.18	3.52	-	1.11	2.61	-	-
**130**	4.84	1.52	2.95	-	0.82	4.63	-	-
**160**	5.78	1.79	2.25	1.82	3.40	1.10	-	-
**200**	6.08	1.55	-	-	10.48	2.45	-	-
**250**	5.52	2.08	16.19	-	19.03	6.51	1.38	
**300**	7.01	2.09	6.36	-	0.99	1.51	-	-

Table S3. Strain variation with Tg , for as grown and annealed ~150nm ZnO films on *a*-Al_2O_3 and *c*-Al_2O_3 substrates.

T_g degC	Strain% (101)_as *a*-Al2O3	Strain% (101)_RTP *a*-Al2O3	Strain% (002)_as *a*-Al2O3	Strain% (002)_RTP *a*-Al2O3	Strain% (002)_as *c*-Al2O3	Strain% (002)_RTP *c*-Al2O3	Strain% (101)_as *c*-Al2O3	Strain% (101)_RTP *c*-Al2O3
**100**	0.28	0.68	0.32	-	0.45	0.27	-	-
**130**	0.38	0.53	0.24	-	0.38	0.07	-	-
**160**	0.89	0.84	0.36	0.73	0.28	0.32	-	-
**200**	0.93	0.64	NA	-	1.11	0.23	-	-
**250**	0.69	0.70	0.16	-	1.13	1.21	4.64	-
**300**	0.97	0.68	0.61	-	0.21	0.32	-	-

bukII_as grown ZnO/c-Al2O3

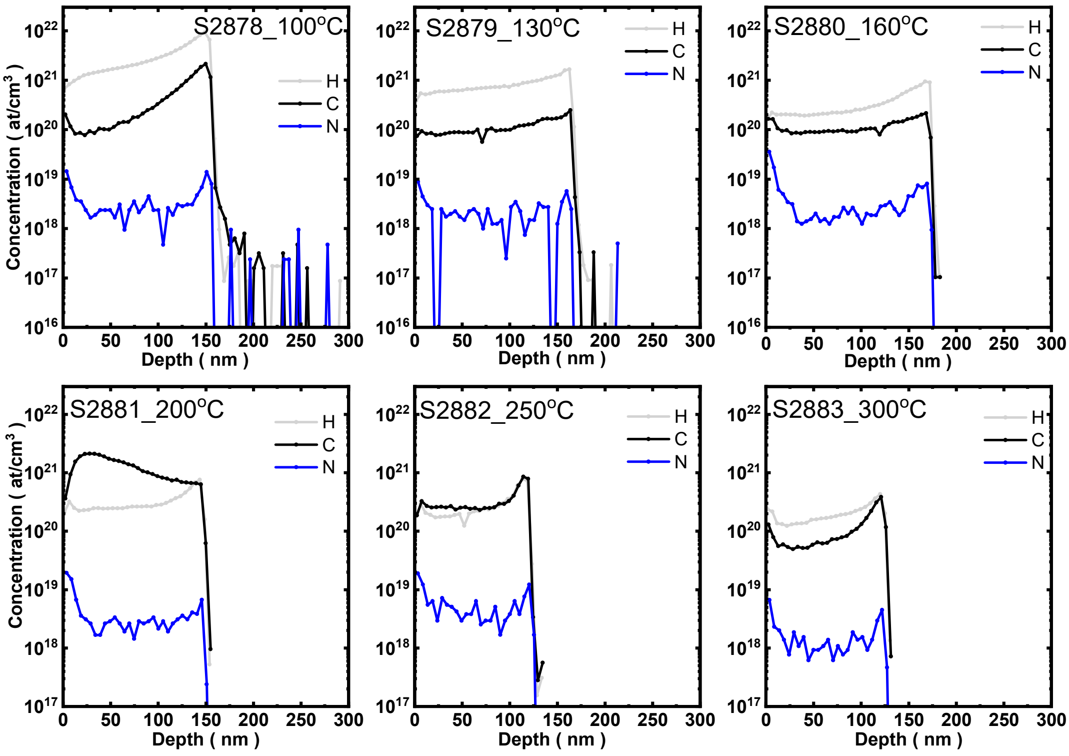

bukII_annealed ZnO/c-Al2O3

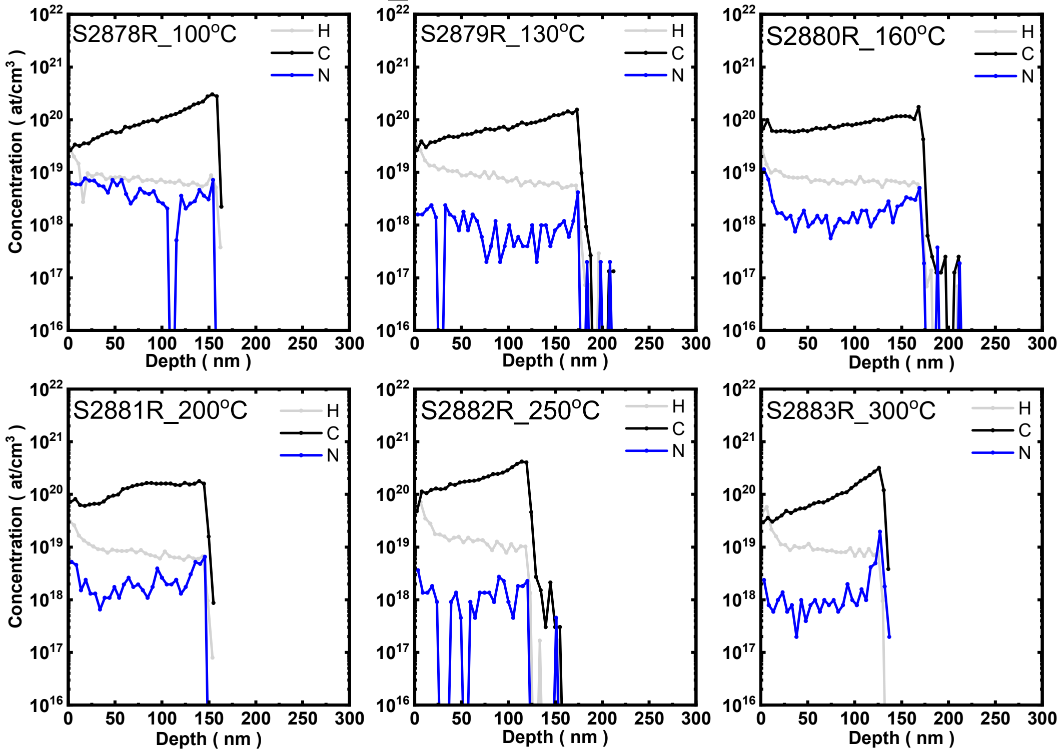

BukII_150nm_as grownZnO/aAl2O3

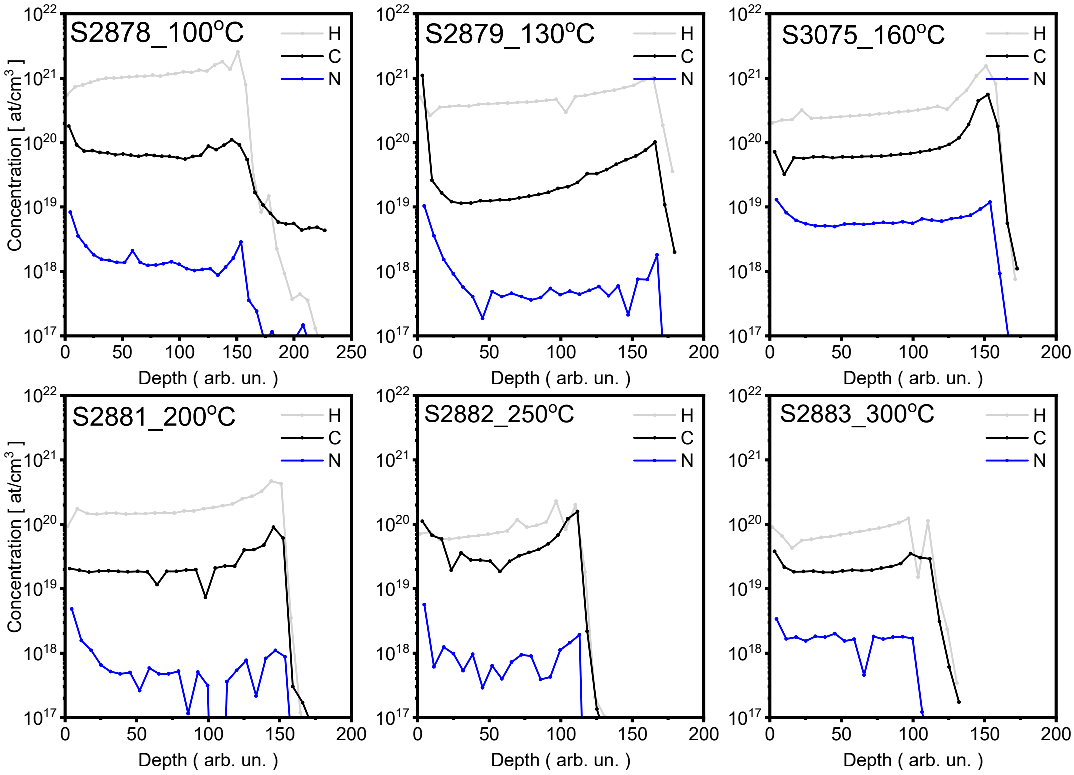

bukII_3minRTPO800_ZnO/*a*-Al2O3

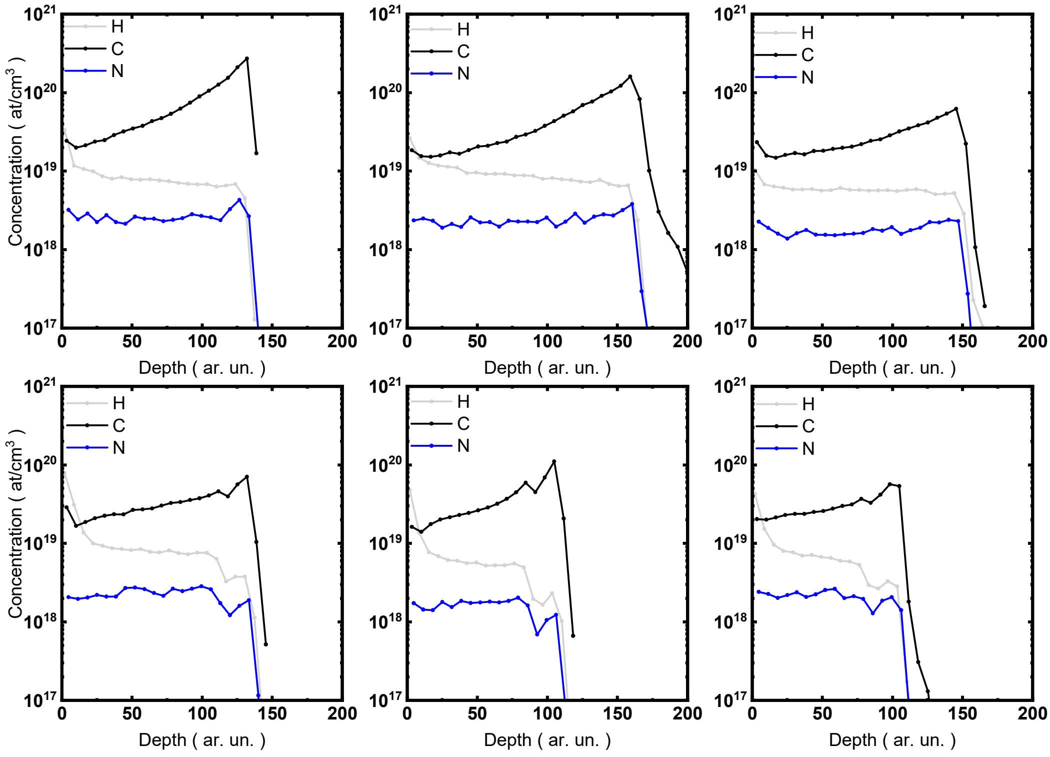

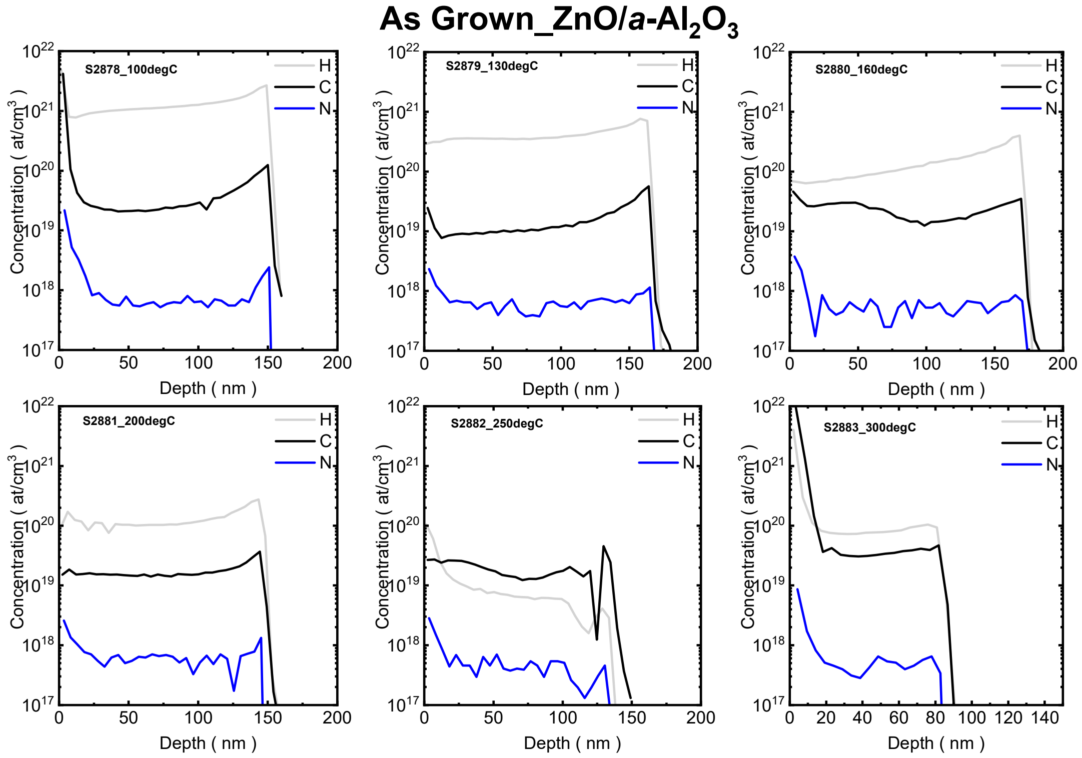

Figure S3. The results of SIMS measurements.

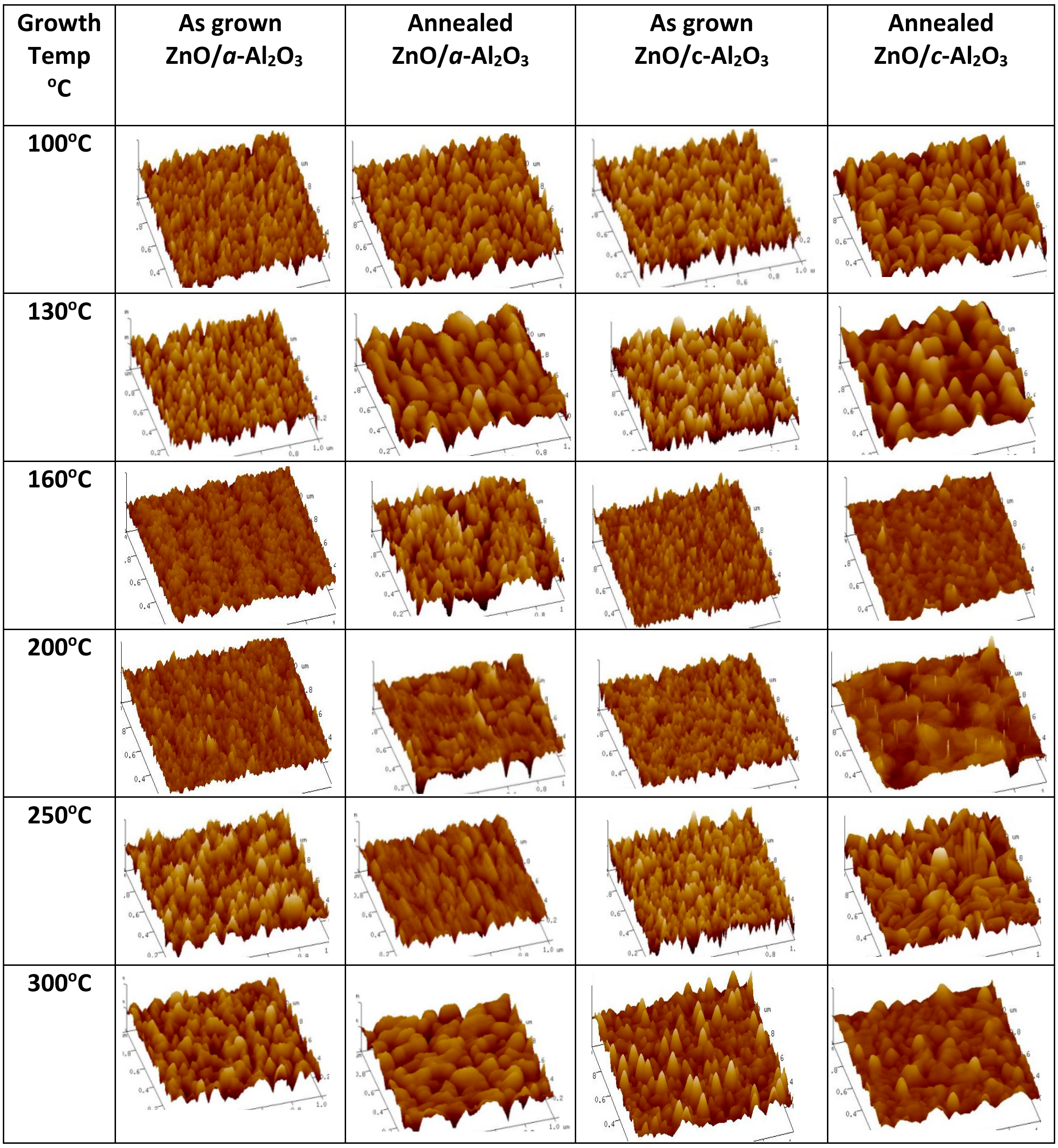

Figure S4. AFM images.

Table S4. The roughness recorded using AFM image.

Growth temperature degC	Roughness(10µm) (Rq) _nm as-ZnO/*c*-Al2O3	Roughness(10µm) (Rq) _nm RTP-ZnO/*c*-Al2O3	Roughness(10µm) (Rq) _nm as-ZnO/*a*-Al2O3	Roughness(10µm) (Rq) _nm RTP-ZnO/*a*-Al2O3
100	6.21	5.1	4.41	5.11
130	7.51	6.01	5.22	4.8
160	2.98	1.25	2.35	2.84
200	1.52	0.968	1.16	1.33
250	2.37	3.74	2.1	2.56
300	8.62	5.93	5.22	4.67
